# Characteristics of Novel Fermented Cloudy Fruit Juices Produced Using *Lactiplantibacillus plantarum* and Lactic Acid-Producing *Lachancea* spp. Yeasts

**DOI:** 10.3390/molecules30193928

**Published:** 2025-09-30

**Authors:** Paweł Satora, Magdalena Skotniczny, Martyna Maziarek

**Affiliations:** Department of Fermentation Technology and Microbiology, Faculty of Food Technology, University of Agriculture, Balicka Street 122, 30-149 Kraków, Poland; magdalena.skotniczny@urk.edu.pl (M.S.);

**Keywords:** functional beverages, pear, plum, polyphenols, volatiles

## Abstract

Fermented fruit juices are considered functional beverages because they contain bioactive compounds derived from plant materials and produced by the microorganisms involved in fermentation. The composition of these beverages can vary depending on the strain used. This study aimed to determine the effect of different microorganisms conducting lactic acid fermentation on the chemical composition and bioactive component content of naturally cloudy fermented pear and plum juices. The process used *Lactiplantibacillus plantarum* K7 bacteria, which were isolated during sauerkraut fermentation, as well as *Lachancea thermotolerans* PYCC6375 and *Lachancea fermentati* PYCC5883 yeast cultures, which have poor ethanol fermentation capabilities. The pH, acidity, sugars (HPLC), free amino nitrogen, selected organic acids (HPLC), color (CIELAB), polyphenols (HPLC), volatiles (GC-MS), aroma-active volatiles (GC-MS-O), and sensory characteristics were analyzed. The fermented juices obtained were rich in organic acids (of plant and microbial origin), polyphenols, and had a reduced sugar content (with polyols replacing glucose and fructose), as well as a low alcohol content (<0.2%). At the same time, all three microorganisms significantly enhanced the fruity aroma of the juices. *Lachancea* yeasts proved to be a viable alternative to lactic acid bacteria for producing fermented juices and were significantly better suited to fermenting plum juices. The highest polyphenol content and highest consumer preference rating were obtained with plum juices fermented with *L. fermentati* yeast.

## 1. Introduction

Pears and plums are popular stone fruits. In 2022, world production amounted to 26,314,506.67 tonnes for pears and 12,564,237.8 tonnes for plums and sloes. Their harvests occupied 1,329,245 hectares and 2,629,126 hectares, respectively. China is the world’s largest producer of both fruits, accounting for over half of global output in 2022 (73% and 55%, respectively). In Poland, however, their harvest and crop area were mainly of regional importance, amounting to 80,600 tonnes and 5500 hectares, and 133,200 tonnes and 16,500 hectares, respectively [1]. Nevertheless, after apples, they are among the most commonly consumed native fruits in Poland. In November 2024, 46% of surveyed Poles declared consuming pears and 31% declared consuming plums [2].

Pears and plums are highly valued fruits due to their high content of bioactive compounds that contribute to their health-promoting properties. However, the composition and concentration of these compounds can vary depending on the species and variety of fruit, its ripeness, and pre- and postharvest conditions [3,4]. Therefore, the region in which the fruit is produced may also play an important role. The dominant bioactive group in these fruits is phenolic compounds, particularly phenolic acids and flavonoids [5,6]. In traditional Eastern medicine, pears (*Pyrus* spp.) have been used to relieve respiratory symptoms, treat fever and inflammation, and alleviate alcohol hangovers [7]. These properties have been confirmed by scientific research, which has also demonstrated their anti-diabetic, anti-obesity, anti-hyperlipidemic, anti-mutagenic, anti-carcinogenic and cardioprotective activity [7]. In turn, plums (*Prunus domestica*) are used in traditional medicine in various regions of the world mainly to support proper digestive function. This has also been confirmed in various scientific studies, which have also demonstrated antioxidant, anti-carcinogenic, anti-hyperlipidemic and anxiolytic activity [8,9]. However, it is worth mentioning that neither fruit may be suitable for people with carbohydrate malabsorption.

The lactic acid fermentation of fruit juices enhances their bioactive properties by releasing beneficial metabolites and increasing the bioavailability of certain nutrients. This also leads to a reduction in sugar content, an extension to the shelf life, and changes to sensory characteristics [10,11]. The effects may vary depending on the strain used. Lactic acid bacteria, such as *Lactiplantibacillus plantarum*, are traditionally involved in this process [12,13,14,15,16,17]. Using unconventional microorganisms instead of these bacteria could lead to new opportunities for developing functional beverages, one of the fastest-growing segments of the food market.

In the research, the non-*Saccharomyces* yeasts belonging to the genus *Lachancea*, which are capable of producing lactic acid were used. So far, they have been used in winemaking for the bioacidification of wine [18,19,20,21] and in brewing for the production of sour and low-alcohol beers [22,23,24,25,26,27]. The *L. thermotolerans* PYCC6375 and *L. fermentati* PYCC5883 strains were selected for the study as they produce high levels of lactic acid and low levels of ethanol during wort fermentation [27]. The aim of the study was to investigate the impact of these strains on the chemical and bioactive properties of naturally cloudy pear and plum juices, compared to lactic acid bacteria. The *L. plantarum* K7 strain used in the experiment was isolated from the sauerkraut fermentation process [28], and previous unpublished experiments have shown that it has excellent properties for fermenting plant material. To date, no such studies have been published in the literature. The idea was to create fermented juices with increased functional potential and minimal sensory changes; therefore, the fermentation was brief, and salt was not added. Such products could gain recognition among increasingly health-conscious consumers.

## 2. Results and Discussion

### 2.1. Fermentation Kinetics and Basic Physicochemical Parameters

**Figure 1:**
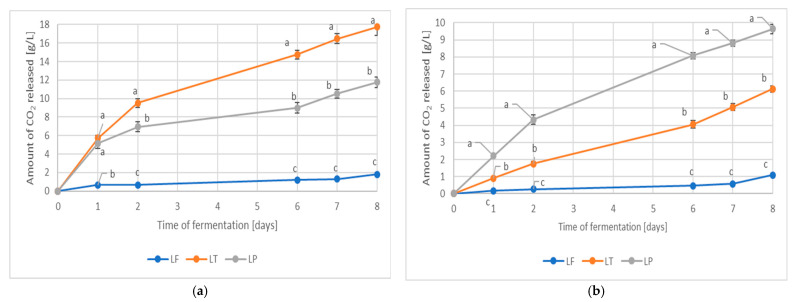
The kinetics of fruit juice fermentation ((**a**) pear juice; (**b**) plum juice) depend on the microorganism used: LP—*Lactiplantibacillus plantarum* K7, LT—*Lachancea thermotolerans* PYCC6375, LF—*Lachancea fermentati* PYCC5883. Values with different Roman letters (a–c) indicate statistically significant differences at *p* < 0.05 according to the Tukey test.

The kinetics of fruit juice fermentation varied depending on the strain and matrix used (Figure 1). In the case of pear juices, the most efficient fermentation was performed by *L. thermotolerans* PYCC6375 (LT), while in the case of plum juices, it was performed by *L. plantarum* K7 (LP). *L. fermentati* PYCC5883 (LF) showed poor fermentation abilities in both types of juice. The same *Lachancea* strains produced similar results during wort fermentation [27].

The growth of microorganisms in juices was possible due to the sufficient amount of sugars and free amino nitrogen (FAN; Table 1). Fructose dominated in pear juice and glucose in plum juice, accounting for approximately 75% and 64% of total sugars, respectively. These results are consistent with previous studies, which state that in addition to glucose and fructose, pears and plums also contain sucrose and sorbitol [29,30]. Glucose was found to be the preferred carbon source for microorganisms in both fruit juices, as evidenced by its higher consumption than fructose. All strains were also able to completely break down sucrose. This may explain why plum juice fermented by the LP strain contained significantly more fructose than unfermented juice. Furthermore, the tested strains were able to produce polyols as a by-product of fermentation. Glycerol appears to be produced most efficiently from glucose. In the case of sorbitol and mannitol, which are isomers, the analytical method used does not allow them to be distinguished. Nevertheless, their concentration in fermented pear juice increased significantly. This suggests that mannitol was formed as a by-product of fructose fermentation [31]. The polyols in fermented juices can compensate for the loss of sweetness caused by the reduction in sugar content. In terms of free amino nitrogen (FAN) compounds, plum juice contained larger amounts than pear juice, reflecting their respective fruit content. The amount of FAN in grapes has been shown to depend on fruit yield and growing conditions, and its concentration may decrease during the storage or processing of the fruit [32]. During fermentation, the tested strains utilized nitrogen compounds, but these differences were not statistically significant. Fermented pear juices had lower FAN values than fermented plum juices, making them more microbiologically stable, since nitrogen is essential for microbial growth.

As is well known, during lactic acid fermentation, microorganisms convert sugars into organic acids, which are the primary by-products. These acids lower the pH and increase the total acidity of the medium. This process is typically performed by lactic acid bacteria. Due to the presence of the lactate dehydrogenase enzyme, *Lachancea* yeast strains also possess this property [22]. All of the tested microorganisms were able to produce organic acids which caused changes in the pH and total acidity of the juices (Table 1). The initial pH of pear juice was 4.5 and of plum juice was 3.3, with average total acidity of 6.17 g/L and 15.25 g/L, respectively. In both juices, all strains significantly reduced the pH to between 3.21 and 3.92 in pear juice and to between 3.07 and 3.11 in plum juice. Concurrently, the total acidity of the fermented juices increased to 6.89–12.9 g/L in pear juice and 18.17–24.12 g/L in plum juice. In both cases, the LP strain proved to be the most efficient. Lowering the pH of juices extends their shelf life and stabilizes their color by preventing browning and inhibiting the activity of polyphenol oxidase, as well as stabilizing plum anthocyanins [33,34].

During fermentation, microorganism produced succinic, lactic and acetic acids, while citric acid was metabolized, but only in pear juice. The level of malic acid did not change after the fermentation of any of the juices. Ethanol was found in all fermented juices, but its content was below 0.2%, which allows the beverages to be classified as non-alcoholic (Table 2). Surprisingly, the LP strain was the most efficient in this respect. During pear juice fermentation, its quantity was higher than that of lactic acid. Ethanol can be produced by *L. plantarum* spp. As a by-product of heterolactic acid fermentation or citrate metabolism, but only in small quantities [35]. Certain strains of *L. plantarum* have also been shown to possess a bifunctional alcohol/aldehyde dehydrogenase gene (AdhE), which converts acetaldehyde to ethanol during mannitol and sorbitol utilization [36,37]. The higher amount of ethanol produced in pear juice may be due to fructose conversion into mannitol via the mannitol dehydrogenase enzyme, which is present in heterofermentative bacteria [38]. Perhaps similar mechanisms occurred in the *Lachancea* strains. Ethanol fermentation in both juices can be confirmed by the increased content of glycerol and, in the case of pear juice, succinic acid. The greater production of ethanol than lactic acid in the fermented pear juice samples suggests that lactic acid fermentation was partially inhibited, probably due to the high fructose content.

As mentioned previously, the type and concentration of organic acids directly influence the pH, but they could also affect the color of the juices. Furthermore, there is a close relationship between color parameters and pigments [39]. The yellow color of pears is mainly due to their carotenoid content, while the purple color of plums is due to their anthocyanin content [40,41]. The results of the CIELab analysis are presented in Table 3. The fermented pear juices were slightly darker (parameter L*) than the control due to an increase in red pigment (parameter a*). This was most likely due to changes in the phenolic composition (phenolic oxidation) [42]. Conversely, the fermented plum juices were lighter than the control due to an increase in yellow pigment (parameter b*). This may be due to the breakdown of anthocyanins forming yellowish compounds [43]. In both juices, the LP strain caused the greatest changes in color (ΔE).

### 2.2. Volatile Compounds

In addition to color, volatile compounds significantly impact the sensory evaluation of the product. Table 4 lists the volatile compounds detected in unfermented and fermented pear and plum juices. Only aroma-active compounds detected by olfactometric analysis and/or exceeding the threshold in any sample are included. These compounds are classified into four groups based on their chemical structure: alcohols, carbonyl compounds, esters, and terpenes.

The aroma of plums is shaped by a complex mixture of volatile compounds, with esters, aldehydes, alcohols, ketones, terpenes and lactones being the most significant contributors [44]. In unfermented plum must, 17 aroma-active components were identified (Table 4). Olfactometric analysis (Table 5) showed that the most pronounced aromas were terpenes, such as rose oxides, (E)-β-damascenone, and β-ionone; six-carbon carbonyl compounds, such as 3-hexenal and hexanal; and ethyl 2-methylbutyrate. These introduced floral (rose), plum, green, and fruity notes. Other compounds typical of plum aroma were also detected in the samples, but at a lower intensity. Key aroma-active components identified across various plum cultivars include esters such as acetic acid hexyl ester, hexyl acetate and butanoic acid butyl ester; aldehydes such as hexanal, (E)-2-hexenal, and 3-hexenal; alcohols such as 1-hexanol and (E)-2-hexen-1-ol; and terpenes such as linalool and limonene [44,45]. Other compounds that contribute significantly to the characteristic ‘plum’ aroma include benzaldehyde, methyl cinnamate, γ-decalactone, and β-damascenone, with the latter being particularly potent in some plum wines [46]. The specific aroma profile varies by plum variety and ripening stage, with certain esters and aldehydes (notably C6 compounds) being closely linked to the perception of fresh, fruity and green notes [44]. Sensory and chemical analyses demonstrate that the diversity and concentration of these volatiles can distinguish between different cultivars and maturity stages, rendering them valuable markers for quality and variety identification [47].

Regardless of the microorganism used, fermentation significantly altered the composition and content of the aroma components. This is particularly noticeable in the case of C6 components, which were not detected in fermented juices. During fermentation, the concentration of C6 compounds—mainly C6 aldehydes (such as hexanal and (E)-2-hexenal) and C6 alcohols (such as 1-hexanol)—typically decreases. This reduction is primarily due to yeast enzymes converting C6 aldehydes into their corresponding alcohols and further metabolic transformations reducing their overall concentration. This transformation is important because C6 compounds contribute green, grassy aromas, and their reduction during fermentation leads to a softer, fruitier aroma profile in the final product. The extent of C6 compound reduction varies depending on fermentation conditions, yeast strain, and the presence of fruit solids or skins [48,49]. A similar phenomenon occurred with most other carbonyl compounds. The content of terpenes derived from the fruit also decreased. However, β-damascenone and β-ionone remained significant components of the aroma of the fermented juice. Terpenes, which are important aroma compounds that contribute floral and fruity notes, often decrease in concentration during fermentation, especially in products such as wine and fruit beverages. This reduction is mainly due to the conversion of glycosidically bound terpenes into free forms, which are then further metabolized, volatilized, or lost through other chemical reactions during fermentation [50]. The extent of terpene loss can vary depending on the fermentation conditions, the species of microorganisms present, and the presence of other compounds in the matrix, such as phenolic acids, which can inhibit terpene hydrolysis and volatilization [51]. Finally, the aroma of fermented plum juices was influenced by the alcohols and esters formed by microorganisms during fermentation. The most important of these were ethyl hexanoate and 3-methyl-1-butanol, which were produced more by yeasts than bacteria. Ethyl hexanoate is a key ester responsible for the fruity aromas found in fermented beverages such as beer, wine, and, in particular, the strongly aromatic Chinese liquor Baijiu. Its formation during fermentation primarily involves the action of yeast, particularly through the alcohol acyltransferase pathway, where enzymes such as EEB1 and Eht1p catalyse the synthesis of ethyl hexanoate from hexanoyl-CoA and ethanol [52]. Lactic acid bacteria (LAB) can synthesize ethyl hexanoate during milk fermentation, mainly through esterification reactions between free fatty acids and ethanol. There is significant variability among strains, with higher activity generally observed for esterification than for alcoholysis [53]. There is little evidence regarding the production of ethyl hexanoate by LAB during the fermentation of fruit juices. Fermenting melon and cashew apple juices with *Lacticaseibacillus casei* resulted in decreased ethyl hexanoate levels, suggesting that LAB may degrade or transform this ester rather than synthesize it in fruit juice matrices [54]. Our research has shown that the LP strain produces similar amounts of ethyl hexanoate to the *Lachancea* sp. Yeast. Due to this compound’s low aroma detection threshold, it significantly influences the aroma of fermented fruit juice. Furthermore, 3-Methyl-1-butanol, also known as isoamyl alcohol, is a key higher alcohol formed during the fermentation of fruit juices that plays a significant role in the aroma of fermented beverages. It is primarily formed through the catabolism of the amino acid leucine via the Ehrlich pathway, whereby yeast and certain bacteria convert leucine into 3-methyl-1-butanol via transamination, decarboxylation and reduction steps [55]. Studies on apple and cherry juice fermentations have identified 3-methyl-1-butanol as a dominant volatile compound in the final product. Its concentration is influenced by the type of fruit, the yeast strain used, and fermentation conditions, such as temperature, pH, and sugar content [56].

The key aroma components of pears are primarily volatile compounds belonging to the ester, aldehyde, and alcohol classes, which together create the fruit’s characteristic fruity and floral scent [57,58]. Esters such as hexyl acetate, butyl acetate, ethyl butanoate and, in particular, ethyl (E,Z)-2,4-decadienoate—often referred to as the ‘pear ester’—are major contributors to the typical pear-like aroma. Ethyl (E,Z)-2,4-decadienoate is especially important in Bartlett pears and pear-based products [59]. Aldehydes such as hexanal and (E)-2-hexenal provide green and fresh notes, while alcohols like 1-hexanol and 2-phenylethanol impart grassy and floral nuances [57,58]. The specific composition and concentration of these volatiles varies widely among pear cultivars and is influenced by factors such as species, ripening stage, and storage conditions [57,58]. In addition to esters, aldehydes and alcohols, smaller amounts of acids, ketones, terpenes and lactones also contribute to the overall aromatic complexity [60]. The balance and abundance of these compounds determine the sensory quality and consumer appeal of different pear varieties [57,58]. The pear musts we examined contained 20 aroma-active components (Table 4 and Table 5). The most important compounds for the pear aroma were C8 compounds such as 1-octen-3-one, 1-octen-3-ol and (E)-2-octenal, C9 and C10 aldehydes—(E)-2-nonenal, decanal, and 2,4-decadienals, as well as terpenes—rose oxides, and β-ionone. As previously mentioned, we detected two esters of 2,4-decadienoic acid, which are characteristic of the pear aroma; the methyl ester was found to have a stronger effect on the aroma than the ethyl ester.

As with plum juice, the aroma of pear juice changed after fermentation, with the strongest aroma found in juices fermented by LP and LT. However, similarly to fresh juice, the presence of C8 components such as 1-octen-3-one, 1-octen-3-ol and (E)-2-octenal was important for the aroma. The components 1-Octen-3-ol and 1-octen-3-one are important volatile compounds known for their characteristic mushroom-like aroma, and their formation is primarily linked to the oxidative breakdown of linoleic acid, with its levels influenced by both the initial composition of the juice and the fermentation matrix [61]. Although there have been few direct studies on these components in fruit juice fermentation, related research on fermented juices (such as kiwifruit and litchi) has shown that fermentation by lactic acid bacteria or yeast can increase the concentrations of various ketones and alcohols, including C8 compounds like 1-octen-3-ol, which is closely related to the formation of 1-octen-3-one [62,63]. The formation of 1-octen-3-one is generally associated with the enzymatic oxidation of linoleic acid and other unsaturated fatty acids during fermentation. Its levels can be affected by the specific strains used, as well as by the presence of glycosidic precursors [61]. (E)-2-Octenal is a volatile aldehyde that is commonly formed during fermentation processes. It contributes to the aroma and flavor profiles of various fermented foods. During juice fermentation, (E)-2-octenal and other aldehydes generally decrease in concentration. In watermelon juice fermented with various lactic acid bacteria, the levels of aldehydes including (E)-2-octenal decreased after fermentation, while alcohols, ketones, acids, and other volatiles increased. This reduction in aldehydes was associated with improved sensory qualities, such as a sweeter and more natural flavor, and a decrease in off-flavors and bitterness. Similarly, lactic acid bacteria fermentation of kiwifruit juice led to a significant decrease in aldehyde concentrations, including (E)-2-octenal, while increasing the levels of esters, ketones and alcohols, which contributed to a more desirable aroma profile [64]. The decrease in (E)-2-octanal during juice fermentation is primarily due to microbial metabolism whereby aldehydes are reduced or transformed into other compounds, resulting in an improvement in the flavor and aroma of the fermented juice [62]. As previously discussed in the context of plum juice, the content of most terpene compounds decreased after fermentation. The exception was β-damascenone, which was not present in the fresh juice but became one of the most important aromatic components after fermentation. Β-Damascenone is a key aroma compound found in pear-derived products. In Bartlett pear brandies, (E)-β-damascenone has been identified as one of the main contributors to the characteristic fruity, pear-like aroma, with high odor activity values indicating its strong sensory impact [65]. The concentration of β-damascenone can change during processing; for instance, fermentation and distillation can influence its levels, enhancing or modifying the overall aroma profile [59]. During grape wine fermentation, damascenone concentrations rise from low or undetectable levels to several parts per billion as fermentation progresses, with further increases observed during barrel ageing. This formation is linked to the transformation of specific ketone precursors by yeast activity, with different yeast strains influencing the amount produced [66]. Various microorganisms, including specific yeast strains, can increase the production of damascenone during fermentation by converting carotenoid-derived precursors into this aromatic compound. The efficiency of damascenone formation depends on the microbial strain and fermentation conditions used, such as pH, sugar content, and fermentation time [67]. As with plum juices, the metabolites produced during fermentation that had the greatest impact on aroma in fermented pear juices were ethyl hexanoate and 3-methyl-1-butanol. Once again, LT produced the greatest quantities of these components, while LF produced the least.

Due to the presence of a buttery aroma in selected fermented juices, the content of volatile organic acids in the samples was also analyzed (Table 4). It was found that this aroma was related to butyric acid, which was produced in quantities greater than the threshold for aroma in pear juice fermented with both *Lachancea* yeast strains. Current research shows that butyric acid production during fermentation is primarily associated with certain bacteria, especially *Clostridium* species, rather than yeasts. These bacteria can utilize C4 compounds (characteristic of pear juices), such as acetate and butyrate precursors, to efficiently generate butyric acid. The metabolic pathway involves converting acetyl-CoA to butyryl-CoA, which is then transformed into butyric acid [68]. These bacteria produce much higher amounts of butyric acid (up to 40 g/L), which is higher than that found in our studies (Table 4). Due to the lack of reports regarding the production of butyric acid by yeasts, this topic is interesting and should be investigated. This is especially important since butyric acid plays several important roles in human health. It serves as a primary energy source for colon cells, supports the maintenance of the intestinal barrier, and has anti-inflammatory effects that can help to protect against conditions such as colitis and other inflammatory bowel diseases [69].

### 2.3. Polyphenols

Both plums and pears are rich sources of phenolic antioxidants, which contribute significantly to their health benefits. The total phenolic content of plums varies widely among cultivars, ranging from 125 to 372.6 mg/100 g, and is strongly correlated with antioxidant capacity as measured by FRAP, TEAC and ORAC assays [70]. The plum skin contains much higher levels of phenolic compounds and flavonoids than the pulp, making it a particularly potent antioxidant source [71]. The major antioxidant compounds found in plums include neochlorogenic acid, chlorogenic acid, anthocyanins (such as cyanidin derivatives), and flavonols such as quercetin [70]. While pears also contain valuable phenolic antioxidants, they generally have lower antioxidant activity than plums. They are rich in hydroxycinnamates, such as caffeic acid and p-coumaric acid, but their overall antioxidant capacity is lower than that of anthocyanin-rich fruits like plums [72].

As shown in Table 6, plum juice contains significantly higher amounts of polyphenols than pear juice. Neochlorogenic acid and its isomer chlorogenic acid were dominant in plums, while the highest amounts of chlorogenic, shikimic, and caffeic acids were found in pear must. Neochlorogenic acid is a major phenolic compound in plums, found in both the skin and pulp but typically in higher concentrations in the skin [73]. It is one of the predominant phenolic compounds in both fresh and dried plums, often exceeding the levels of related compounds such as chlorogenic acid [74]. The content of neochlorogenic acid can vary depending on plum variety, the stage of fruit development, and even the rootstock used for cultivation [75]. Its concentration generally decreases as the fruit matures, with higher levels observed in less ripe plums. Chlorogenic acid is a major phenolic compound found in both plums and pears, though its concentration is typically much higher in plums. In plums, chlorogenic acid and its isomer neochlorogenic acid are both abundant, contributing significantly to the total phenolic content. Some studies recommend using chlorogenic acid as a standard for measuring phenolic compounds in chlorogenic acid-rich plum cultivars [76]. In pears, chlorogenic acid also plays a role in enzymatic browning and fruit quality. The content of chlorogenic acid in pears decreases as the fruit matures, and its synthesis is regulated by specific genes, with higher levels sometimes found in dwarf pear varieties [77]. The presence of chlorogenic acid in both fruits is associated with antioxidant activity and potential health benefits, such as reducing oxidative stress and providing anxiolytic effects [78].

Shikimic acid is a naturally occurring organic acid found in pears and plums. While shikimic acid itself is not classified as a polyphenol, it is a key metabolite in the shikimate pathway, which plays a crucial role in the biosynthesis of numerous plant polyphenols and phenolic compounds [79]. In pears, shikimic acid is consistently identified as one of the main organic acids, although its concentration is generally lower than that of malic and citric acids. Studies of various pear cultivars demonstrate that shikimic acid content can fluctuate depending on the species, cultivar, and developmental stage and is positively correlated with other acids, such as quinic acid [80]. In plums, shikimic acid is also present as part of the organic acid profile. Its content typically increases during early fruit development and then decreases as the fruit ripens [81]. Shikimic acid has several documented influences on human health, including antioxidant, anti-inflammatory, antibacterial, and analgesic effects. It has been shown to suppress lipid accumulation and reduce the expression of genes involved in fat synthesis, suggesting a potential role in preventing or treating fatty liver disease and hyperlipidemia [82].

Juice fermentation increased the amount of analyzed polyphenolic and related compounds, depending on the type of microorganism used (Table 6). The best results were obtained with the yeast *L. fermentati*, which was particularly evident in plum juices fermented with this microorganism. The juices used for fermentation were naturally cloudy, especially the plum juices, and contained a large number of solid particles. It is assumed that substances with antioxidant activity were released from these particles during fermentation with the participation of microbiological enzymes. Fermentation often leads to an increase in polyphenol concentration in various foods and beverages. This increase is primarily due to the release of bound polyphenols and the transformation of polyphenolic compounds by microbial enzymes, which makes them more bioavailable and enhances their antioxidant activity [83]. However, the effect can vary depending on the food matrix and fermentation conditions. For example, some polyphenols may decrease while others increase during apple cider fermentation [84]. Taking into account the dominant polyphenols in the tested juices (Table 6), research shows that chlorogenic acid concentration can either increase or decrease after fermentation, depending on the substrate, fermentation conditions, and microorganisms used. Studies on blueberry and blackberry juices fermented with various lactic acid bacteria and probiotics report a significant reduction in chlorogenic acid levels following fermentation, as the compound is metabolized by microorganisms or transformed into other phenolic compounds [85]. For instance, chlorogenic acid often disappeared or decreased in cider apple juice and mixed fruit juices during fermentation, while other acids such as quinic acid increased, suggesting microbial metabolism of chlorogenic acid [86]. In solid-state fermentation of soybeans with *L. casei*, the yield of chlorogenic acid was significantly higher in the fermented samples than in the unfermented ones. This indicates that fermentation can enhance the extraction and availability of chlorogenic acid by breaking down cell wall components and releasing bound phenolics [87].

### 2.4. Sensory Analysis

**Figure 2:**
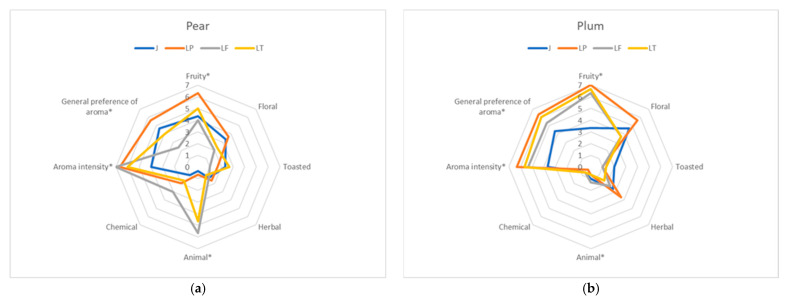
Spider web diagrams showing the aroma qualities and impressions of unfermented (J) pear (**a**) and plum (**b**) juices and of juices produced using different fermentation microorganisms: LP—*Lactiplantibacillus plantarum* K7, LT—*Lachancea thermotolerans* PYCC6375, LF—*Lachancea fermentati* PYCC5883). Parameters that are statistically significantly different according to a two-way ANOVA (*p* < 0.05) are marked with an asterisk (*).

**Figure 3:**
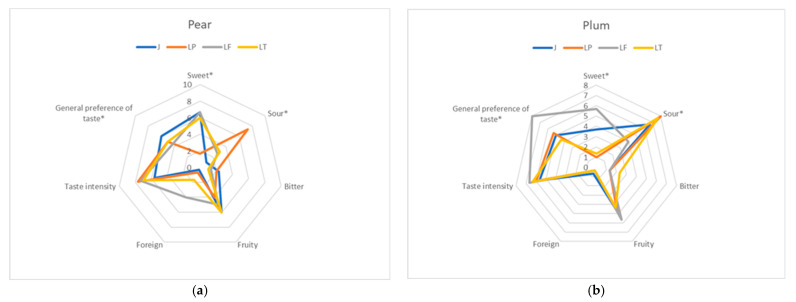
Spider web diagrams showing the taste qualities and impressions of unfermented (J) pear (**a**) and plum (**b**) juices and of juices produced using different fermentation microorganisms: LP—*Lactiplantibacillus plantarum* K7, LT—*Lachancea thermotolerans* PYCC6375, LF—*Lachancea fermentati* PYCC5883). Parameters that are statistically significantly different according to a two-way ANOVA (*p* < 0.05) are marked with an asterisk (*).

The research concluded with a sensory analysis, during which the aroma and taste of both unfermented and fermented musts were assessed (Figure 2 and Figure 3). The aroma and taste of various samples of pear juice showed significantly greater variability than those of plum juice. At the same time, unfermented juices had a lower aroma intensity and were rated lower than fermented juices. Using all three microorganisms significantly enhanced the fruity aroma of the juices. This was due not only to the creation of new components, such as esters [53], but also to the conversion of compounds in the fruit from bound (non-volatile) to free (volatile) forms [50]. Juices fermented with the LP strain were rated highest in terms of aroma (Figure 2). The only drawback of pear juices fermented with *Lachancea* yeast was their buttery flavor, which resulted from higher levels of butyric acid, as mentioned. This lowered the overall rating of these juices. However, butyric acid has also been reported to have positive effects on the human body [69]. No sensory evidence of butyric acid was found in plum juices; therefore, all fermented juices were rated equally highly.

In terms of taste, juice scores were most strongly influenced by sourness (related to the production of lactic acid) and sweetness (related to the use of sugars during fermentation). Research consistently shows that sourness can suppress the perception of sweetness in juices and other beverages. Studies using both human panels and biohybrid taste systems demonstrate that as the concentration of sour compounds (such as citric and tartaric acids) increases, the perceived sweetness of sugars decreases, and vice versa—sweetness can also suppress perceived sourness [88]. Therefore, the juices fermented with LAB had the strongest sour taste and were the least sweet. Yeast positively contributed to increasing the intensity of the juices’ taste. In the case of pear juices, the juice fermented with the LF strain was rated the lowest (mainly due to a foreign aftertaste, probably related to the presence of butyric acid), whereas plum juices received the highest scores by far. This juice was characterized by a balanced feeling of sweetness and sourness, and a strong, fruity plum flavor.

## 3. Materials and Methods

### 3.1. Microbial Strains and Media Used

The *Lactiplantibacillus plantarum* K7 strain used in the research was isolated from sauerkraut and is deposited in the collection of pure cultures of the Department of Fermentation Technology and Microbiology, University of Agriculture in Krakow, Poland. The yeasts *Lachancea thermotolerans* PYCC6375 and *Lachancea fermentati* PYCC5883 were obtained from the Portuguese Yeast Culture Collection (PYCC), Caparica, Portugal. Microorganisms were cultivated on MRS Broth (LAB; Biomaxima, Lublin, Poland) or Sabouraud Dextrose Broth (yeasts; Biomaxima, Lublin, Poland).

### 3.2. Preparation and Fermentation of Juices

The Conference variety of pears (*Pyrus communis* L.) and the Węgierka zwykła variety of plums (*Prunus domestica* L.) were purchased from a local market in Kraków, Poland. The fruit was washed in boiling water, dried, cut into quarters, and juiced using a slow juicer (ZC420E38; Tefal, Rumilly, France) to obtain naturally cloudy juice. Three hundred milliliters of juice were poured into sterile fermentation flasks and pasteurized in a water bath (AJL electronic, Krakow, Poland) at 90 °C for ten minutes.

The juices were aseptically inoculated with a final concentration of 10^8^ CFU/mL of lactic acid bacteria or selected yeast strains. The number of bacteria was determined using a densitometer (Biosan, Jozefow, Poland) based on a standard curve developed for the LAB strain. The number of yeast cells was determined through direct counting using a Thom chamber. The flasks were then closed with rubber stoppers and fitted with fermentation tubes filled with glycerol. For each variety, assays were performed in triplicate. Fermentation was conducted for eight days at 22 °C. Weight losses were measured daily to monitor the process. Once fermentation was complete, the samples were centrifuged at 179× *g* (MPW-350R; CONBEST, Krakow, Poland) and then pasteurized at 85 °C for 20 min.

### 3.3. pH and Acidity Measurement

The pH of the juices was measured using a CP-505 pH meter (Elmetron, Zabrze, Poland). The total acidity of the juices was determined by potentiometric titration of 25 mL of the sample with 0.1 N NaOH until a pH of 7 was reached.

### 3.4. Organic Acids’ Analysis

The organic acids were analyzed using a UV–Vis detector at a wavelength of 210 nm on a NEXERA XR apparatus (Shimadzu, Kyoto, Japan), with the REZEX ROA-Organic Acid H+ (8%) Phenomenex (USA) column. The juices were centrifuged, and the resulting supernatant was filtered using a 0.45 μm syringe filter. The samples were diluted fivefold with distilled water. Concentrations of the individual components were determined using previously prepared standard curves for citric, malic, succinic, lactic, and acetic acids (all Sigma-Aldrich, St. Louis, MO, USA).

### 3.5. Sugar and Ethanol Analysis

Sugar compounds and ethanol were analyzed using a Shimadzu NEXERA XR apparatus (Kyoto, Japan) fitted with an RF-20A refractometric detector and a Shodex Asahipak NH2P-50 4.6 × 250 mm column (Showa Denko Europe, Munich, Germany), which was thermostatted at 30 °C. The mobile phase was a 70% aqueous acetonitrile solution, and the isocratic elution programme lasted 16 min at a flow rate of 0.8 mL/min. Concentrations of the individual components were determined based on standard curves previously prepared for glucose, fructose, sucrose, glycerol, sorbitol and ethanol (all Sigma-Aldrich, St. Louis, MO, USA).

### 3.6. Free Amino Nitrogen Analysis

Free amino nitrogen (FAN) was quantified using the ninhydrin method. Ninhydrin reacts with NH_3_ upon exposure to heat to form a colored complex. Therefore, the ninhydrin reagent was added to water (blank), the test samples, and the reference sample (glycine standard). The samples were then boiled for 10 min, and the absorbance was measured at 575 nm using a DU-650 UV–Vis spectrophotometer (Beckman, Brea, CA, USA).

### 3.7. Color Analysis

The color of the juices was measured according to the CIELab method using a CM-3500d colorimeter (Konica Minolta, Inc., Tokyo, Japan). A D65 light source and an observation angle of 10° were used. The following parameters were measured: L^-^ (brightness) (L^-^ = 0 black, L* = 100 white), a* (proportion of green) (a* < 0) or red (a* > 0), b* (proportion of blue) (b* < 0) or yellow (b* > 0), C* (color saturation) and h* (color angle). Unfermented juices were compared with fermented juices. In order to determine the color difference between the samples, the parameter ΔE was calculated from the following formula:

$$\Delta E=\surd ({〖(\Delta L)〗}^{2}+{〖(\Delta a)〗}^{2}+{〖(\Delta b)〗}^{2}).$$

### 3.8. Volatile Compounds’ Analysis (HS-SPME-GC-MS)

To analyse the volatile compounds, 0.05 g of juice, 2 mL of distilled water, and 0.1 mL of an internal standard solution (containing 0.57 mg/L of 4-methyl-2-pentanol, 0.2 mg/L of anethol and 1.48 mg/L of ethyl nonanoate, all from Sigma-Aldrich, St. Louis, MO, USA) were placed in a 10 mL vial containing 1 g of NaCl. A conditioned (250 °C for 1 h) SPME device (Supelco Inc., Bellefonte, PA, USA) coated with 100 μm PDMS fibers was used for sampling. This was placed in the headspace, with stirring at 300 rpm for 40 min at 40 °C. Next, the SPME device was desorbed in the injector port of the chromatograph system for 3 min. These analyses were performed using a Shimadzu GC-2010 Plus gas chromatograph coupled with a GCMS-QP2020 system. Separation of the analyzed volatiles was performed using a Rxi^®^-1 ms capillary column (Crossbond 100% dimethyl polysiloxane; 30 m × 0.53 mm × 0.5 μm). The column was heated using the following programme: 35 °C for 4 min, increasing by 5 °C/min to 110 °C; then increasing by 20 °C/min to 230 °C; then maintaining a constant temperature for 4 min. Helium was used as the carrier gas at a constant flow rate of 1.0 mL/min. The analyte was transferred in splitless mode. Mass spectra were recorded in SEM mode. Compound identification was performed using mass spectral libraries and linear retention indices derived from the C6 to C30 n-alkane series. Volatiles (Sigma-Aldrich) were identified qualitatively and quantitatively by comparing the retention times and peak areas of the sample and standard chromatograms. The results obtained were analyzed using the NIST database.

### 3.9. Odor-Active Volatile Components (HS-SPME-GC-O)

The odor-active volatile compounds in the juices were identified by olfactometry. A 2 mL sample of beer containing 1 g of NaCl was placed in a 10 mL vial and exposed to a divinylbenzene/carboxen/polydimethylsiloxane (DVB/CAR/PDMS) SPME fibre (50/30 µm, Supelco/Sigma-Aldrich, Bellafonte, PA, USA) for 40 min at 40 °C. The SPME device was then introduced into the injector port of the Hewlett Packard 5890 Series II chromatograph system and left there for 3 min. The components were then separated using an Rxi-1 ms capillary column (Crossbond 100% dimethyl polysiloxane, 30 m × 0.53 mm × 0.5 µm). The detector temperature was set to 250 °C, and the column was heated using the following programme: 35 °C for four minutes at an increment of 5 °C/min up to 110 °C, followed by an increment of 20 °C/min up to 230 °C, and then maintaining a constant temperature for four minutes. The carrier gas was helium at a constant flow of 1.0 mL/min. For analysis, an olfactory detection port (ODP-3, Gerstel, Linthicum, MD, USA) was used. A trained GC-O analyst was asked to describe the perceived odors and their intensity. Odor intensity was assessed using a 4-point scale (not detected, weak, moderate, and strong).

### 3.10. Determination of Volatile Organic Acids by Derivatization with Benzyl Bromide

One milliliter of the sample was transferred into the reaction vial, along with one milliliter of distilled water, one milliliter of buffer and 15 microliters of benzyl bromide. The sample was stirred at 50 °C for a defined period of time (210 min). After derivatization, the vial was cooled in an ice water bath, and the solution was saturated with 1 g of NaCl. It was then thermostatted at 30 °C and the analyte extracted by headspace SPME using a PA fibre (85 µm, Supelco Inc., Bellefonte, PA, USA) for 60 min. Next, the SPME device was desorbed in the injector port of the chromatograph system for 3 min. A Shimadzu GC-2010 Plus gas chromatograph coupled with a GCMS-QP2020 system was used for the analysis. An Rxi^®^-1 ms capillary column (Crossbond 100% dimethyl polysiloxane, 30 m × 0.53 mm × 0.5 µm) was used to separate the analyzed volatiles. The initial temperature was 40 °C for 5 min. It was then raised to 250 °C at a rate of 4 °C per minute. The transfer line temperature was 250 °C, and the mass spectrometer was operated in electron impact mode (70 eV). Helium was used as the carrier gas at a constant flow rate of 1.0 mL/min. Mass spectra were recorded in EI mode at an ionization voltage of 70 eV, with a transfer line and ion source temperature of 250 °C. Standard curves were prepared using volatile acids (butyric, hexanoic, octanoic, decanoic, dodecanoic; Sigma-Aldrich) by derivatization under the conditions described above.

### 3.11. Polyphenol Analysis

Polyphenol analysis was performed using a Shimadzu Nexera XR system (Shimadzu, Kyoto, Japan), which consists of a CBM-20A system controller, an LC-20AD VP pump, an SIL-10AC VP autoinjector, a DGU-20A degasser, an SPD-M20A VP UV–VIS detector and LabSolutions ver. 5.93, as well as a Shim-pack VP-ODS 250 × 4.6 (5 µm) column. The injection volume was 10 μL. Solutions were filtered through a 0.45 µm nylon membrane prior to HPLC injection. A reverse-phase HPLC assay was carried out using a gradient system with a flow rate of 0.7 mL/min and a column temperature of 25 °C. The mobile phase consisted of phosphoric acid at a concentration of 0.1% (solution A) and acetonitrile (solution B). The elution programme was as follows: a linear increase in the concentration of solution B from 14% to 19% over 8 min; isocratic elution with 19% solution B over 6 min; a linear increase in the concentration of solution B from 19% to 31% over 20 min; a linear increase in the concentration of solution B from 31% to 90% over 5 min; a linear decrease in the concentration of solution B to 14% over 1 min; and isocratic elution with 14% solution B over 9 min. Shikimic acid was detected at λ = 210 nm; vanillic acid at λ = 250 nm; salicylic acid, gallic acid, ellagic acid, and catechin at λ = 280 nm; neochlorogenic acid, chlorogenic acid, caffeic acid, gentisic acid, p-coumaric acid, and ferulic acid at λ = 325 nm; quercetin at λ = 360 nm; and cyanidin-3-O-glucoside and cyanidin-3-O-rutinoside at λ = 520 nm.

Standard curves were prepared using the appropriate standards for quantitative determinations: shikimic acid, vanillic acid, salicylic acid, ellagic acid, (+)-catechin, neochlorogenic acid, chlorogenic acid, caffeic acid, gentisic acid, ferulic acid, quercetin, cyanidin-3-O-glucoside and cyanidin-3-O-rutinoside (all from Sigma-Aldrich), gallic acid, p-coumaric acid, and ferulic acid (all from Extrasynthese, Genay, France).

### 3.12. Sensory Analysis (QDA)

A sensory assessment of juice samples was performed using quantitative descriptive analysis (QDA). The evaluation was conducted by ten trained testers (five men and five women) from the Department of Fermentation Technology and Microbiology, aged between 30 and 50 years. Fifteen sensory qualities, selected in an initial panel discussion, were evaluated on a ten-point scale; these related to aroma (fruity, floral, toasted, herbal, animal, chemical, intensity, general preference) and taste (sweet, sour, bitter, fruity, foreign, intensity, general preference).

### 3.13. Statistical Analysis

All analyses were performed in triplicate, and their results are presented in the paper as arithmetic means with standard deviation. Statistical analysis of pH, acidity, content of individual components, and juice color was performed using a two-way analysis of variance (ANOVA) using Tukey’s HSD post hoc test for *p* < 0.05 (Statistica, v. 13.3; StatSoft Inc., Tulsa, OK, USA).

## 4. Conclusions

The food market is looking for new natural products with the highest possible health-promoting value and sensory properties that consumers will find acceptable. Such products may be fruit juices fermented with various microorganisms (without added salt) that contain microbiological metabolites and living microbial cells, as well as components derived from plant material such as polyphenols, organic acids, and volatile compounds. In our research, we used cloudy pear and plum juices, which are widely consumed in various parts of the world in their natural state; lactic acid bacteria derived from plant fermentation; and lactic acid-producing yeasts belonging to the genus *Lachancea*. The resulting juices contained high levels of health-promoting compounds (polyphenols were more bioavailable), lower levels of sugars (which were metabolized during fermentation), and potentially toxic substances (such as ethanol). They also had modified sensory characteristics which were often rated higher than unfermented juices. *Lachancea* yeast proved to be a viable alternative to lactic acid bacteria, producing similar quantities and qualities of components, and being resistant to low pH. They performed well in juices that naturally contained high levels of organic acids. However, further research into using these microorganisms to ferment fruit musts with significant acidity (such as blackcurrant and cherry)—in order to soften their taste, stabilize them, and introduce their own characteristic components—is required. The practical nature of our research is also significant, as it provides a basis for product recipes that can be implemented in production and brought to market, thereby increasing the range of beverages that have a positive impact on our health.

## Figures and Tables

**Table 1:** The content of sugar, polyols, and free amino nitrogen (FAN) in unfermented and fermented pear and plum juices depending on the microorganism used.

Fruit Juice	Sample	Glucose	Fructose	Sucrose	Glycerol	Sorbitol + Mannitol	FAN
		[g/L]					
Pear	J	29.7 ^d^ ± 0.5	98.4 ^e^ ± 1.6	3.7 ^b^ ± 1.2	0.00 ^a^ ± 0.00	31.4 ^b^ ± 2.0	65.4 ± 1.8
	LP	4.5 ^b^ ± 0.4	45.6 ^cd^ ± 5.4	0.0 ^a^ ± 0.0	4.26 ^b^ ± 0.68	39.8 ^c^ ± 3.3	24.6 ± 8.9
	LT	1.2 ^a^ ± 0.3	37.5 ^bcd^ ± 5.0	0.0 ^a^ ± 0.0	4.82 ^bc^ ± 1.70	43.4 ^c^ ± 1.1	15.1 ± 2.2
	LF	4.0 ^b^ ± 0.2	48.3 ^d^ ± 4.7	0.0 ^a^ ± 0.0	5.99 ^bc^ ± 0.74	39.1 ^c^ ± 0.4	36.9 ± 10.4
Plum	J	69.9 ^e^ ± 0.1	27.6 ^ab^ ± 0.8	11.7 ^c^ ± 0.8	0.00 ^a^ ± 0.00	30.7 ^ab^ ± 0.3	165 ± 2.9
	LP	9.9 ^c^ ± 0.8	36.5 ^bc^ ± 3.1	0.0 ^a^ ± 0.0	9.05 ^d^ ± 1.12	26.9 ^ab^ ± 0.4	129 ± 20.3
	LT	4.4 ^b^ ± 0.7	18.1 ^a^ ± 4.8	0.0 ^a^ ± 0.0	7.62 ^cd^ ± 1.56	25.1 ^a^ ± 1.6	134 ± 25.0
	LF	4.8 ^b^ ± 1.5	19.6 ^a^ ± 2.1	0.0 ^a^ ± 0.0	6.21 ^bcd^ ± 0.78	27.8 ^ab^ ± 3.9	120 ± 21.0
Sig.	Fruit juice	***	***	***	***	***	***
	Fermentation	***	***	***	***	ns	***
	Fruit juice × fermentation	***	***	***	**	***	ns

Legend: J—unfermented juice, LP—*Lactiplantibacillus plantarum* K7, LT—*Lachancea thermotolerans* PYCC6375, LF—*Lachancea fermentati* PYCC5883; Sig.—significance; ns—not significant. ** and *** indicate significance at levels of 0.01–0.005 and <0.005, respectively, using the least significant difference test. Values with different superscript Roman letters (a–e) in the same column indicate statistically significant interactions between fruit juice and fermentation (Tukey HSD, *p* < 0.05).

**Table 2:** The pH, total acidity, organic acids, and ethanol content of unfermented and fermented pear and plum juices depending on the microorganism used.

Fruit Juice	Sample	pH	Total Acidity	Citric Acid	Malic Acid	Succinic Acid	Lactic Acid	Acetic Acid	Ethanol
			[g/L]						
Pear	J	4.50 ^a^ ± 0.02	6.17 ± 0.29	1.91 ^a^ ± 0.21	3.64 ± 0.19	0.23 ^a^ ± 0.16	0.00 ^a^ ± 0.00	0.00 ^a^ ± 0.00	0.00 ^a^ ± 0.00
	LP	3.21 ^c^ ± 0.02	12.9 ± 2.36	1.54 ^b^ ± 0.25	3.49 ± 0.37	1.18 ^b^ ± 0.75	0.29 ^ab^ ± 0.02	1.50 ^c^ ± 0.21	1.32 ^e^ ± 0.29
	LT	3.92 ^b^ ± 0.06	6.84 ± 2.00	0.80 ^b^ ± 0.87	2.79 ± 0.27	0.88 ^b^ ± 0.96	0.96 ^b^ ± 0.33	1.32 ^bc^ ± 0.06	1.14 ^de^ ± 0.20
	LF	3.81 ^c^ ± 0.06	7.08 ± 1.70	1.30 ^b^ ± 0.66	3.01 ± 0.47	1.82 ^c^ ± 0.30	0.45 ^ab^ ± 0.12	1.02 ^b^ ± 0.29	0.31 ^ab^ ± 0.06
Plum	J	3.30 ^c^ ± 0.03	15.25 ± 0.43	3.13 ^c^ ± 0.11	2.35 ± 0.12	0.02 ^a^ ± 0.01	0.00 ^a^ ± 0.00	0.00 ^a^ ± 0.00	0.00 ^a^ ± 0.00
	LP	3.07 ^d^ ± 0.01	24.12 ± 0.62	3.14 ^c^ ± 0.21	2.32 ±0.66	0.31 ^a^ ± 0.03	2.71 ^c^ ± 0.37	0.07 ^a^ ±0.04	0.86 ^cd^ ± 0.21
	LT	3.11 ^d^ ± 0.00	18.17 ± 0.29	3.27 ^c^ ± 0.09	2.30 ± 0.68	0.27 ^a^ ± 0.05	2.79 ^c^ ± 0.57	0.14 ^a^ ± 0.04	0.36 ^ab^ ± 0.09
	LF	3.10 ^d^ ± 0.02	20.52 ± 4.38	3.21 ^c^ ± 0.32	2.36 ± 0.20	0.29 ^a^ ± 0.02	2.25 ^c^ ± 0.39	0.13 ^a^ ± 0.07	0.45 ^bc^ ± 0.04
Sig.	Fruit juice	***	***	***	***	***	***	***	***
	Fermentation	***	***	**	ns	***	***	***	***
	Fruit juice × fermentation	***	ns	***	ns	***	***	***	***

Legend: J—unfermented juice, LP—*Lactiplantibacillus plantarum* K7, LT—*Lachancea thermotolerans* PYCC6375, LF—*Lachancea fermentati* PYCC5883; Sig.—significance; ns—not significant. ** and *** indicate significance at levels of 0.01–0.005 and <0.005, respectively, using the least significant difference test. Values with different superscript Roman letters (a–e) in the same column indicate statistically significant interactions between fruit juice and fermentation (Tukey HSD, *p* < 0.05).

**Table 3:** CIELab color parameters (L*, a*, b*, C*, h*) and color differences (ΔE) in unfermented and fermented pear and plum juices depending on the microorganism used.

Fruit Juice	Sample	L^-^	a^-^	b^-^	C^-^	h*	ΔE
Pear	J	70.9 ^d^ ± 1.9	11.6 ± 0.8	55.5 ^bc^ ± 1.7	56.7 ^a^ ± 1.8	78.2 ^d^ ± 0.5	
	LP	63.8 ^cd^ ± 4.0	24.0 ± 2.1	64.6 ^d^ ± 1.5	69.0 ^cd^ ± 0.7	69.6 ^c^ ± 2.1	15.7 ± 2.4
	LT	68.9 ^d^ ± 5.0	17.4 ± 2.5	58.8 ^cd^ ± 6.7	61.4 ^ab^ ± 6.8	73.5 ^cd^ ± 2.1	9.4 ± 2.1
	LF	64.6 ^cd^ ± 2.5	19.7 ± 2.2	62.5 ^d^ ± 1.4	65.5 ^bc^ ± 1.8	72.6 ^c^ ± 1.6	10.8 ± 3.1
Plum	J	55.8 ^b^ ± 2.6	35.0 ± 3.5	62.4 ^d^ ± 1.5	71.7 ^de^ ± 0.7	60.7 ^b^ ± 3.0	
	LP	39.8 ^a^ ± 1.5	46.5 ± 2.2	47.4 ^a^ ± 1.6	66.4 ^bcd^ ± 2.0	45.5 ^a^ ± 1.7	22.3 ± 2.2
	LT	43.6 ^a^ ± 2.9	46.2 ± 2.6	52.0 ^ab^ ± 3.4	69.6 ^cd^ ± 1.9	48.4 ^a^ ± 3.2	17.0 ± 4.9
	LF	57.4 ^bc^ ± 4.5	41.7 ± 6.0	64.5 ^d^ ± 3.1	77.0 ^e^ ± 1.1	57.1 ^b^ ± 5.0	9.4 ± 2.8
Sig.	Fruit juice	***	***	***	***	***	
	Fermentation	***	***	***	***	***	
	Fruit juice × fermentation	***	ns	***	***	***	

Legend: J—unfermented juice, LP—*Lactiplantibacillus plantarum* K7, LT—*Lachancea thermotolerans* PYCC6375, LF—*Lachancea fermentati* PYCC5883; Sig.—significance; ns—not significant. *** indicates significance at a level of <0.005 using the least significant difference test. Values with different Roman numerals (a–e) in the same column indicate statistically significant interactions between fruit juice and fermentation (Tukey HSD, *p* < 0.05).

**Table 4:** The volatile compounds in unfermented and fermented pear and plum juices depending on the microorganism used.

Compound [μg/L]	*m*/*z*	LRI	THR	Pear Juice				Plum Juice				Sig.		
				J	LP	LT	LF	J	LP	LT	LF	Fruit Juice	Fermentation	Fruit Juice x Fermentation
**Alcohols**														
3-Methyl-1-butanol	42, 55, 70	724	71	2 ^a^	111 ^ab^	358 ^cd^	256 ^bc^	2 ^a^	341 ^cd^	410 ^cd^	422 ^d^	***	***	*
(Z)-3-Heksen-1-ol	41, 55, 67	837	13.0	1.5 ^a^	0.1 ^a^	0.2 ^a^	0.1 ^a^	30.1 ^b^	3.3 ^a^	3.3 ^a^	3.2 ^a^	***	***	***
Heksan-1-ol	41, 43, 56	852	100	297 ^c^	45 ^a^	50 ^a^	41 ^a^	185 ^b^	29 ^a^	28 ^a^	27 ^a^	ns	***	***
1-Heptanol	41, 56, 70	953	5.4	0.0 ^a^	4.4 ^bc^	7.8 ^c^	7.7 ^c^	0.0 ^a^	3.5 ^ab^	2.3 ^ab^	0.8 ^ab^	***	***	***
1-Octen-3-ol	43, 57, 72	961	0.01 ^1^ 0.5 ^2,3^	5.0 ^bc^	7.1 ^cde^	9.4 ^e^	8.4 ^de^	0.2 ^a^	5.9 ^bcd^	3.9 ^bc^	3.0 ^ab^	***	***	***
**Carbonyl compounds**														
2-Methylbutanal	42, 55, 86	640	1.0	0.0 ^a^	0.0 ^a^	0.0 ^a^	0.0 ^a^	2.3 ^b^	0.0 ^a^	0.0 ^a^	0.0 ^a^	***	***	***
3-Hexenal	41, 55, 69	773	0.25	0.0 ^a^	0.0 ^a^	0.0 ^a^	0.0 ^a^	9.4 ^b^	0.0 ^a^	0.0 ^a^	0.0 ^a^	***	***	***
Hexanal	41, 44, 56	776	10.0	287 ^c^	0.0 ^a^	1.0 ^a^	1.0 ^a^	160 ^b^	0.0 ^a^	0.0 ^a^	0.0 ^a^	***	***	***
2-Hexenal	41, 55, 69	825	17.0	44.5 ^c^	0.0 ^a^	0.0 ^a^	0.0 ^a^	25.1 ^b^	0.0 ^a^	0.0 ^a^	0.0 ^a^	***	***	***
1-Octen-3-one	55, 70, 97	954	0.01 ^1^ 0.5 ^2,3^	2.2 ^b^	2.6 ^b^	3.6 ^c^	2.1 ^b^	0.0 ^a^	0.2 ^a^	0.3 ^a^	0.5 ^a^	***	***	***
Octanal	44, 56, 81	978	0.7	0.0 ^a^	1.1 ^d^	0.9 ^c^	0.2 ^b^	0.3 ^b^	0.0 ^a^	0.0 ^a^	0.0 ^a^	***	***	***
Phenylacetaldehyde	65, 91, 120	1005	2.0	0.3 ^a^	0.1 ^a^	0.1 ^a^	0.1 ^a^	12.0 ^b^	0.1 ^a^	0.1 ^a^	0.2 ^a^	***	***	***
(E)-2-Octenal	41, 55, 70	1036	0.2	6.7 ^c^	0.9 ^b^	1.7 ^b^	0.9 ^b^	0.0 ^a^	0.2 ^a^	0.2 ^a^	0.3 ^a^	***	***	***
Nonanal	41, 55, 70	1084	1.0	3.8 ^b^	0.4 ^a^	0.5 ^a^	0.3 ^a^	3.4 ^b^	0.3 ^a^	0.2 ^a^	0.2 ^a^	ns	***	ns
(E)-2-Nonenal	41, 55, 70	1134	0.1	3.0 ^b^	0.1 ^a^	0.2 ^a^	0.1 ^a^	0.0 ^a^	0.0 ^a^	0.0 ^a^	0.0 ^a^	***	***	***
Decanal	41, 55, 70	1181	0.1	1.0 ^b^	0.2 ^a^	0.2 ^a^	0.1 ^a^	1.5 ^c^	0.0 ^a^	0.1 ^a^	0.0 ^a^	***	***	ns
(Z,Z)-2,4-Decadienal	41, 67, 81	1264	0.2	4.5 ^b^	0.0 ^a^	0.0 ^a^	0.0 ^a^	0.0 ^a^	0.0 ^a^	0.0 ^a^	0.0 ^a^	***	***	***
(E,E)-2,4-Decadienal	41, 67, 81	1284	0.4	3.2 ^b^	0.0 ^a^	0.0 ^a^	0.0 ^a^	0.0 ^a^	0.0 ^a^	0.0 ^a^	0.0 ^a^	***	***	***
(E)-2-Undecenal	41, 57, 70	1332	0.8	0.0 ^a^	0.4 ^b^	1.0 ^d^	0.7 ^c^	0.0 ^a^	0.0 ^a^	0.0 ^a^	0.0 ^a^	***	***	***
**Esters**														
Ethyl butyrate	43, 71, 88	785	1.0	0.0 ^a^	0.6 ^ab^	0.6 ^ab^	0.8 ^bc^	0.0 ^a^	1.4 ^c^	0.8 ^bc^	0.7 ^abc^	ns	***	ns
Ethyl L-lactate	43, 45, 75	795	50.0	0.0 ^a^	63.3 ^c^	65.0 ^c^	59.2 ^c^	0.0 ^a^	56.3 ^bc^	52.6 ^bc^	32.2 ^b^	**	***	ns
Butyl acetate	43, 56, 73	798	10.0	2.6 ^ab^	16.5 ^d^	3.7 ^b^	12.4 ^c^	1.4 ^ab^	0.0 ^a^	0.0 ^a^	0.0 ^a^	***	***	***
Ethyl 2-methylbutyrate	57, 85, 102	836	0.1	0.5 ^a^	0.1 ^a^	0.3 ^a^	0.1 ^a^	1.4 ^b^	0.3 ^a^	0.4 ^a^	0.2 ^a^	**	***	***
Ethyl isovalerate	41, 57, 102	838	0.1	0.0 ^a^	0.0 ^a^	0.1 ^b^	0.0 ^a^	0.0 ^a^	0.4 ^c^	0.4 ^c^	0.4 ^c^	***	***	***
Isoamyl acetate	55, 43, 70	860	12.0	0.0 ^a^	27.2 ^c^	12.4 ^b^	15.3 ^b^	0.0 ^a^	18.5 ^bc^	16.3 ^b^	10.0 ^b^	ns	***	*
2-Methylbutyl acetate	43, 55, 70	862	5.0	0.0 ^a^	1.4 ^abc^	2.5 ^abc^	1.2 ^ab^	0.0 ^a^	5.2 ^c^	4.7 ^bc^	3.2 ^abc^	***	***	ns
Ethyl hexanoate	43, 88, 99	979	1.0	0.3 ^a^	3.5 ^b^	5.4 ^b^	3.6 ^b^	0.3 ^a^	5.5 ^b^	5.1 ^b^	3.3 ^ab^	ns	***	ns
Hexyl acetate	43, 56, 61	992	2.0	5.7 ^e^	2.2 ^d^	1.1 ^bc^	1.3 ^c^	0.5 ^ab^	0.0 ^a^	0.0 ^a^	0.0 ^a^	ns	***	***
Methyl salicylate	92, 120, 152	1170	35	31.3 ^b^	0.1 ^a^	0.1 ^a^	0.1 ^a^	83.2 ^c^	2.7 ^a^	2.4 ^a^	5.0 ^a^	***	***	***
Methyl (E,Z)-2,4-decadienoate	67, 81, 111	1367	0.1	0.8 ^c^	0.1 ^ab^	0.2 ^b^	0.1 ^ab^	0.0 ^a^	0.0 ^a^	0.0 ^a^	0.0 ^a^	***	***	***
Ethyl decanoate	70, 88, 101	1370	5.0	3.1 ^ab^	4.3 ^bc^	3.6 ^abc^	3.2 ^ab^	0.9 ^a^	6.1 ^cd^	7.6 ^d^	3.6 ^abc^	***	***	*
Ethyl (E,Z)-2,4- decadienoate	67, 81, 125	1434	0.1	0.6 ^b^	0.1 ^a^	0.0 ^a^	0.0 ^a^	0.0 ^a^	0.0 ^a^	0.0 ^a^	0.0 ^a^	**	***	***
**Terpenes and others**														
Acetal	45, 47, 73	721	4.9	0.0 ^a^	1.8 ^a^	6.5 ^ab^	9.4 ^b^	0.0 ^a^	20.6 ^c^	9.3 ^b^	4.6 ^ab^	***	***	***
Linalool	43, 71, 93	1086	1.0	4.4 ^c^	0.7 ^ab^	1.2 ^ab^	0.4 ^a^	4.1 ^c^	1.4 ^b^	1.3 ^ab^	1.1 ^ab^	**	***	ns
(Z)-Rose oxide	55, 69, 139	1095	0.1	77.4 ^c^	0.1 ^a^	0.1 ^a^	0.1 ^a^	45.8 ^b^	0.2 ^a^	0.1 ^a^	0.1 ^a^	**	***	***
(E)-Rose oxide	55, 69, 139	1113	0.1	34.6 ^c^	0.1 ^a^	0.1 ^a^	0.1 ^a^	20.4 ^b^	0.1 ^a^	0.1 ^a^	0.1 ^a^	**	***	***
(E)-β-Damascenone	69, 105, 121	1365	0.002 ^1^ 0.1 ^2,3^	0.0 ^a^	1.5 ^bc^	0.4 ^a^	0.6 ^ab^	2.6 ^cd^	2.7 ^d^	2.6 ^d^	2.3 ^cd^	***	*	***
α-Ionone	43, 93, 121	1411	0.4	0.5 ^b^	0.0 ^a^	0.0 ^a^	0.0 ^a^	0.3 ^ab^	0.0 ^a^	0.0 ^a^	0.0 ^a^	ns	***	ns
β-Ionone	43, 73, 177	1475	0.007	1.9 ^b^	0.1 ^a^	0.1 ^a^	0.1 ^a^	1.4 ^b^	0.1 ^a^	0.1 ^a^	0.1 ^a^	ns	***	ns
**Volatile organic acids [μg/L]**														
Butyric acid	41, 60, 73	780	10.0	0.0 ^a^	7.3 ^b^	15.7 ^c^	32.7 ^d^	0.0 ^a^	0.0 ^a^	0.0 ^a^	3.4 ^ab^	***	***	***
Hexanoic acid	41, 60, 73	962	36.0	6.5 ^c^	1.7 ^a^	3.2 ^a^	6.8 ^c^	2.1 ^a^	5.5 ^bc^	4.5 ^bc^	6.6 ^c^	ns	*	***
Octanoic acid	43, 60, 73	1162	910	17.5 ^b^	4.4 ^a^	5.9 ^a^	7.4 ^a^	26.9 ^c^	4.5 ^a^	6.7 ^a^	8.8 ^a^	*	***	*
Decanoic acid	41, 60, 73	1339	130	6.8 ^b^	1.6 ^ab^	1.4 ^a^	30.8 ^d^	13.1 ^c^	2.0 ^ab^	2.6 ^ab^	2.2 ^ab^	***	***	***
Dodecanoic acid	43, 60, 73	1530	7200	1.9 ^bc^	0.0 ^a^	5.2 ^d^	4.4 ^d^	2.5 ^c^	1.8 ^bc^	1.3 ^b^	1.2 ^ab^	***	***	***

Legend: LRI—linear retention index, THR—threshold, ^1^—value for unfermented juices, ^2^—value for fermented pear juice, ^3^—value for fermented plum juice, J—unfermented juice, LP—*Lactiplantibacillus plantarum* K7, LT—*Lachancea thermotolerans* PYCC6375, LF—*Lachancea fermentati* PYCC5883, Sig.—significance; ns—not significant. *, **, and *** indicate significance at levels of 0.05–0.01, 0.01–0.005, and <0.005, respectively, as determined by the least significant difference test. Values with different superscript Roman letters (a–e) in the same row indicate statistically significant interactions between fruit juice and fermentation (Tukey HSD, *p* < 0.05). Green color indicates a concentration above the threshold.

**Table 5:** The aroma-active components of unfermented and fermented pear and plum juices depend on the microorganism used.

Compound	LRI	Pear Juice				Plum Juice				Aroma
		J	LP	LT	LF	J	LP	LT	LF	
2-Methylbutanal	640	0	0	0	0	1.5	0	0	0	malty fermented
Acetal	721	0	0	0.5	1	0	2	1	0.5	ether green
3-Methyl-1-butanol	724	0	1	2	2	0	2	2	2	fusel alcoholic
3-Hexenal	773	0	0	0	0	2.5	0	0	0	leafy green
Hexanal	776	2.5	0	0	0	2.5	0	0	0	green aldehydic
Butyric acid	780	0	0	0.5	1.5	0	0	0	0.5	buttery cheesy
Ethyl butyrate	785	0	0	0	0	0	0.5	0	0	fruity pineapple
Ethyl L-lactate	795	0	0.5	0.5	0.5	0	0.5	0.5	0	fruity buttery
Butyl acetate	798	0	1	0	0.5	0	0	0	0	fruity banana
2-Hexenal	825	1.5	0	0	0	0.5	0	0	0	almond green
Ethyl 2-methylbutyrate	836	2	0.5	1.5	0.5	2.5	1.5	2	1	apple fruity
(Z)-3-Heksen-1-ol	837	0	0	0	0	1.5	0	0	0	green grassy
Ethyl isovalerate	838	0	0	0.5	0	0	2	2	2	fruity apple
Heksan-1-ol	852	1.5	0	0	0	1	0	0	0	pungent green
Isoamyl acetate	860	0	1.5	0.5	0.5	0	1	0.5	0	fruity banana
2-Methylbutyl acetate	862	0	0	0	0	0	0.5	0.5	0	sweet banana
1-Heptanol	953	0	0	0.5	0.5	0	0	0	0	herbal green
1-Octen-3-one	954	3	2	2.5	2	0	0	0	0.5	herbal mushroom
1-Octen-3-ol	960	3	2.5	2.5	2.5	2.5	2.5	2.5	2	mushroom earthy
Octanal	978	0	1	0.5	0	0	0	0	0	aldehydic citrus
Ethyl hexanoate	979	0	2	2	2	0	2	2	2	fruity apple
Hexyl acetate	992	1.5	0.5	0	0	0	0	0	0	fruity green apple
Phenylacetaldehyde	1005	0	0	0	0	2	0	0	0	green floral
(E)-2-Octenal	1035	2.5	2	2.5	2	0	0.5	0.5	0.5	cucumber green
Nonanal	1084	2	0	0	0	2	0	0	0	aldehydic rose
Linalool	1086	2	0	0.5	0	2	0.5	0.5	0.5	citrus floral
(Z)-Rose oxide	1095	3	0.5	0.5	0.5	3	1	0.5	0.5	red rose
(E)-Rose oxide	1113	3	0.5	0.5	0.5	3	0.5	0.5	0.5	herbal
(E)-2-Nonenal	1134	2.5	0.5	1	0.5	0	0	0	0	green cucumber
Methyl salicylate	1170	0	0	0	0	1.5	0	0	0	wintergreen mint
Decanal	1181	2.5	1	1	0.5	2.5	0	0.5	0	aldehydic orange peel
(Z,Z)-2,4-Decadienal	1264	2.5	0	0	0	0	0	0	0	seaweed
(E,E)-2,4-Decadienal	1284	2.5	0	0	0	0	0	0	0	oily cucumber
(E)-2-Undecenal	1332	0	0	0.5	0	0	0	0	0	fruity citrus
(E)-β-Damascenone	1365	0	2.5	2	2	3	2.5	2.5	2.5	sweet plum
Methyl (E,Z)-2,4- decadienoate	1367	2.5	0.5	1	0.5	0	0	0	0	fruity pear
Ethyl decanoate	1370	0	0	0	0	0	0.5	1	0	fruity apple brandy
α-Ionone	1411	0.5	0	0	0	0	0	0	0	woody floral
Ethyl (E,Z)-2,4- decadienoate	1434	2	0.5	0	0	0	0	0	0	green pear
β-Ionone	1475	3	2.5	2.5	2.5	3	2.5	2.5	2.5	floral woody

Legend: LRI—linear retention index, J—unfermented juice, LP—*Lactiplantibacillus plantarum* K7, LT—*Lachancea thermotolerans* PYCC6375, LF—*Lachancea fermentati* PYCC5883. The lowest values in the row are colored dark red, while the highest values are colored dark green.

**Table 6:** The polyphenol and related compound contents of unfermented and fermented pear and plum juices depending on the microorganism used.

Compound [mg/L]	Pear Juice				Plum Juice				Sig.		
	J	LP	LF	LT	J	LP	LF	LT	Fruit Juice	Fermentation	Fruit Juice × Fermentation
**Phenolic acids**											
Neochlorogenic acid	0.00 ^a^	0.00 ^a^	0.00 ^a^	0.00 ^a^	129 ^b^	371 ^c^	946 ^d^	458 ^c^	***	***	***
Chlorogenic acid	3.09 ^a^	1.90 ^a^	5.02 ^a^	1.50 ^a^	13.37 ^a^	36.09 ^b^	91.25 ^c^	45.10 ^b^	***	***	***
Caffeic acid	2.22 ^bc^	0.88 ^a^	1.14 ^ab^	0.72 ^a^	0.06 ^a^	0.37 ^a^	2.76 ^c^	0.82 ^a^	ns	***	***
Vanillic acid	0.25 ^a^	1.46 ^c^	2.93 ^d^	1.59 ^c^	0.34 ^a^	0.55 ^ab^	4.23 ^e^	1.08 ^bc^	ns	***	***
Salicylic acid	1.72 ^ab^	0.99 ^a^	0.98 ^a^	1.39 ^ab^	2.43 ^b^	1.63 ^ab^	4.74 ^c^	4.24 ^c^	***	***	***
Gentisic acid	0.05 ^ab^	0.01 ^a^	0.05 ^ab^	0.02 ^ab^	0.07 ^bc^	0.06 ^bc^	0.22 ^d^	0.11 ^c^	***	***	***
Gallic acid	0.96 ^ab^	0.20 ^ab^	0.25 ^ab^	0.00 ^a^	8.94 ^d^	2.45 ^bc^	12.18 ^e^	3.96 ^c^	***	***	***
Ferulic acid	0.02 ^a^	0.00 ^a^	0.00 ^a^	0.00 ^a^	0.00 ^a^	0.39 ^b^	0.34 ^b^	1.39 ^c^	***	***	***
p-Coumaric acid	0.49 ^b^	0.00 ^a^	0.00 ^a^	0.00 ^a^	0.17 ^a^	0.24 ^ab^	0.97 ^c^	1.07 ^c^	***	***	***
Ellagic acid	0.00 ^a^	0.00 ^a^	0.00 ^a^	0.00 ^a^	1.26 ^e^	0.31 ^b^	1.00 ^d^	0.67 ^c^	***	***	***
**Flavonoids**											
Catechin	0.31 ^a^	0.46 ^a^	0.61 ^a^	0.41 ^a^	6.87 ^a^	22.52 ^b^	69.16 ^c^	27.34 ^b^	***	***	***
Quercetin	1.40 ^bc^	1.31 ^abc^	1.46 ^bc^	0.97 ^ab^	1.70 ^c^	1.64 ^c^	1.46 ^bc^	0.73 ^a^	ns	**	***
**Anthocyanins**											
Cyanidin-3-O-rutinoside	0.71 ^c^	0.00 ^a^	0.00 ^a^	0.00 ^a^	0.65 ^bc^	0.13 ^a^	0.75 ^c^	0.45 ^b^	***	***	***
Cyanidin-3-O-glucoside	1.08 ^ab^	0.97 ^ab^	1.56 ^c^	1.00 ^ab^	0.74 ^a^	1.12 ^ab^	1.26 ^bc^	1.22 ^bc^	ns	***	***
**Others**											
Shikimic acid	361 ^d^	141 ^b^	201 ^c^	247 ^c^	48 ^a^	7 ^a^	10 ^a^	9 ^a^	***	***	***

Legend: J—unfermented juice, LP—*Lactiplantibacillus plantarum* K7, LT—*Lachancea thermotolerans* PYCC6375, LF—*Lachancea fermentati* PYCC5883, Sig.—significance; ns—not significant. ** and *** indicate significance at levels of 0.01–0.005 and <0.005, respectively, as determined by the least significant difference test. Values with different Roman letters (a–e) in the same row indicate statistically significant interactions between fruit juice and fermentation (Tukey HSD, *p* < 0.05).

## Data Availability

The original contributions presented in this study are included in the article. Further inquiries can be directed to the corresponding authors.

## Abbreviations

The following abbreviations are used in this manuscript:

J	Raw juice
LP	Juice fermented by *Lactilantibacillus plantarum* K7
LT	Juice fermented by *Lachancea thermotolerans* PYCC6375
LF	Juice fermented by *Lachancea fermentati* PYCC5883
